# Regulation of Met1‐linked polyubiquitin signalling by the deubiquitinase OTULIN

**DOI:** 10.1111/febs.13547

**Published:** 2015-11-02

**Authors:** Paul R. Elliott, David Komander

**Affiliations:** ^1^ Medical Research Council Laboratory of Molecular BiologyCambridgeUK

**Keywords:** deubiquitinase, LUBAC, Met1‐linked, NF‐κB, OTULIN, signalling, substrate‐assisted catalysis, ubiquitin

## Abstract

Modification of proteins with Met1‐linked ‘linear’ ubiquitin chains has emerged as a key regulatory signal to control inflammatory signalling via the master regulator, the transcription factor nuclear factor κB (NF‐κB). While the assembly machinery, the linear ubiquitin chain assembly complex (LUBAC), and receptors for this ubiquitin chain type have been known for years, it was less clear which deubiquitinating enzymes (DUBs) hydrolyse Met1 linkages specifically. In 2013, two labs reported the previously unannotated protein FAM105B/OTULIN to be this missing Met1 linkage‐specific DUB. Structural studies have revealed how OTULIN achieves its remarkable specificity, employing a mechanism of ubiquitin‐assisted catalysis in which a glutamate residue on the substrate complements the active site of the enzyme. The specificity of OTULIN enables it to regulate global levels of Met1‐linked polyubiquitin in cells. This ability led to investigations of NF‐κB activation from new angles, and also revealed involvement of Met1‐polyubiquitin in Wnt signalling. Interestingly, OTULIN directly interacts with LUBAC, and this interaction is dynamic and can be regulated by OTULIN phosphorylation. This provides a new paradigm for how individual linkage types can be regulated by dedicated enzyme complexes mediating assembly and removal. Here we review what has been learned about OTULIN's mechanism, regulation and function, discuss the open questions in the field, and discuss how DUBs regulate the NF‐κB response.

**Abbreviations**
CYLD: cylindromatosis tumour suppressor
DUB: deubiquitinating enzyme
Dvl2: Dishevelled‐2
FADD: Fas‐associated protein with death domain
HECT: homologous to E6‐AP C terminus
HOIL‐1L: haem‐oxidised IRP2 ubiquitin ligase 1L
HOIP: HOIL‐1L interacting protein
IAP: inhibitor of apoptosis protein
IKK: IκB kinase
IκB: inhibitor of κB
LDD: linear ubiquitin chain determination domain
LUBAC: linear ubiquitin chain assembly complex
NEMO: NF‐κB essential modifier
NF‐κB: nuclear factor κB
NOD: nucleotide binding and oligomerisation domain
NZF: Npl4‐like zinc finger
OTU: ovarian tumour
PIM: PUB‐interaction motif
PUB: peptide:N‐glycanase/UBA‐ or UBX‐containing proteins
RBR: RING in‐between RING
RING: really interesting new gene
RIPK1: receptor‐interacting serine/threonine kinase 1
SHARPIN: SHANK‐associated RH domain‐interacting protein
TLR: Toll‐like receptor
TNF: tumour necrosis factor
TRADD: TNFR1‐associated death domain
TRAF: TNF receptor‐associated factor
UBAN: ubiquitin binding in ABIN and NEMO
UBA: ubiquitin association
UBD: ubiquitin binding domain
UBL: ubiquitin‐like
USP: ubiquitin‐specific protease
XIAP: X‐linked inhibitor of apoptosis protein

## Introduction

Cell survival depends on adaptation to the environment, and is mediated by numerous signalling cascades that translate extrinsic signals into an appropriate response. Signalling is most commonly propagated through specific, orchestrated and rapid post‐translational modifications of signalling proteins, often resulting in changes at the transcriptional and translational level. However, if cells cannot adapt or suffer severe damage, signalling cascades activate controlled cell death, apoptosis or necroptosis. Due to these important roles in balancing cellular life‐and‐death decisions, signalling pathways are tightly controlled, and their dysfunction can cause cancer, diabetes, as well as inflammatory and neurodegenerative diseases.

Protein ubiquitination is a highly versatile post‐translational modification intricately involved in most known cellular processes and many signalling cascades. The prevalent outcome of ubiquitin modification is the targeted destruction of proteins by the proteasome 1, but ubiquitination serves many additional roles in degradative and non‐degradative processes 2.

During ubiquitination, ubiquitin is attached to Lys residues in protein substrates by an ATP‐driven cascade involving E1, E2 and E3 enzymes 1, 3. Ubiquitinated proteins are recognised by receptors that contain ubiquitin binding domains (UBDs) and transmit the ubiquitin signal 4. Ubiquitination is reversible, and specialised deubiquitinases (DUBs) hydrolyse ubiquitin linkages and antagonise ubiquitin signalling 5. More than 600 E3 ligases facilitate ubiquitination of thousands of substrates, hundreds of proteins contain UBDs and ~ 80 active DUBs reverse the ubiquitin signal 6.

The ubiquitin signal is highly complex. Proteins can be modified by a single ubiquitin molecule on one or multiple Lys residues (mono/multi‐monoubiquitination). Additionally, ubiquitin can be ubiquitinated, on seven internal lysine residues and on the amino terminus, resulting in polyubiquitin chain formation. Such chains can contain a single linkage type (homotypic chains) or multiple linkage types (heterotypic chains), whereby polymers can be mixed (extension of one homotypic chain with another) or branched (one ubiquitin modified at multiple sites) 2.

Importantly, the type of ubiquitination often determines the fate of the modified protein. Lys48‐ and Lys11 linkages are found on proteins that are degraded, while non‐degradative ubiquitin signals most commonly involve Lys63‐ and Met1‐linked polyubiquitin. Cellular roles of other ‘atypical’ linkage types are still unclear 7, 8.

**Figure 1:**
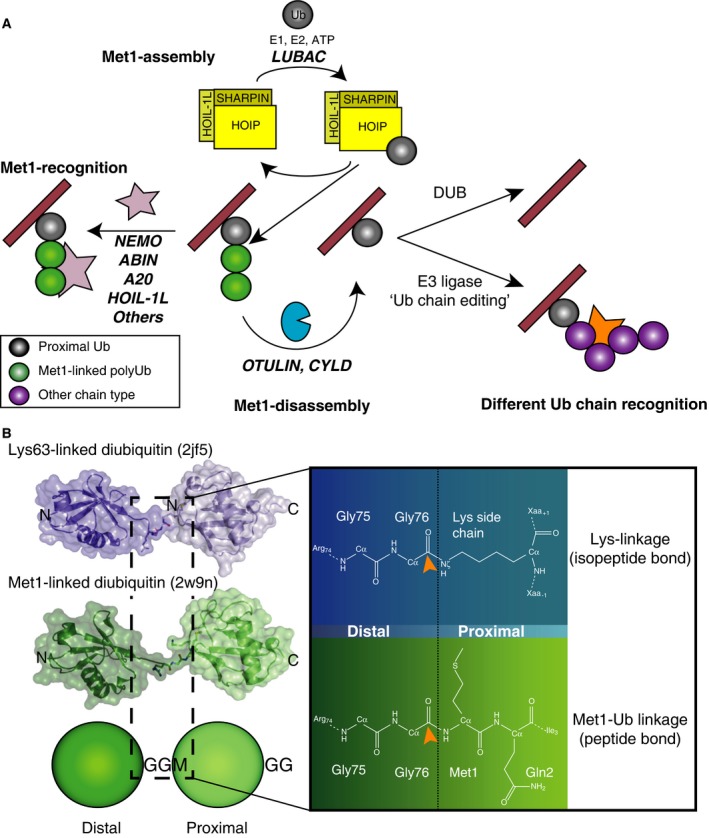
The nature of the Met1 polyubiquitin signal. (A) Met1‐linked polyubiquitin generation, recognition and hydrolysis is facilitated by linkage‐specific proteins. LUBAC may require pre‐ubiquitinated substrates for Met1‐chain assembly, and similarly, OTULIN is unable to remove the proximal ubiquitin from a substrate due to its mechanism (see Fig. 2). Either OTULIN products can be further deubiquitinated by other DUBs, or chain types can be replaced by E3 ligases (‘ubiquitin chain editing’) involving alternative processes, such as degradation. (B) Structures of Lys63‐ (cyan) and Met1‐linked (green) diubiquitin and the schematic representation of the Met1 linkage for distal and proximal ubiquitin moieties. Inset: the chemical differences of the isopeptide and peptide bonds. Arrows point to the electrophilic carbonyl of the scissile bond.

The distinct fates of ubiquitinated proteins suggested that the proteins in the ubiquitin system can be highly linkage specific and indeed, many linkage‐specific proteins have now been reported. The impact and independence of distinct chain types is exemplified by recent discoveries surrounding the Met1‐linked (or linear) polyubiquitin chains (Fig. 1A).

Until a decade ago, Met1‐linked chains were only known to be the translation product of the polyubiquitin genes *UBB* and *UBC*. This changed with discovery of the first, and to date only, Met1 linkage‐specific E3 ligase system, the linear ubiquitin chain assembly complex (LUBAC), by Iwai and colleagues 9. LUBAC is formed from HOIL1‐interacting protein, RNF31 (HOIP), haem‐oxidised IRP2 ubiquitin ligase 1L (HOIL‐1L; RBCK1) and Shank associated RH‐domain interacting protein (SHARPIN) 10, but the stoichiometry of components is unclear. Both HOIP and HOIL‐1L belong to the really interesting new gene (RING) in‐between RING (RBR) family of E3 ubiquitin ligases 11. HOIP exclusively assembles Met1‐linked polyubiquitin, utilising a unique ubiquitin binding region C‐terminal to the RBR, the linear ubiquitin chain determination domain (LDD), that orients the proximal ‘acceptor’ ubiquitin moiety for peptide bond formation towards the distal ‘donor’ ubiquitin moiety 12, 13, 14.

In addition to LUBAC, several Met1 linkage‐specific UBDs have been reported, predominantly in proteins involved in inflammatory pathways. These include the ubiquitin binding in ABIN and NEMO (UBAN) domains of inhibitor of κB kinase (IKK)‐γ also known as NF‐κB essential modifier (NEMO) 15, 16 and other proteins, as well as zinc‐binding UBDs in A20 17, 18 and HOIL‐1L 19 (Fig. 1A).

LUBAC is an important mediator of inflammatory signalling cascades and regulates the activation of the transcription factor nuclear factor‐κB (NF‐κB) by numerous stimuli 20, 21, 22, 23, 24. This is in part based on genetic evidence from studies in mice 22, 23, 24 as well as the occurrence of mutations in LUBAC components in human inflammatory conditions 25, 26. Physiological roles of LUBAC and Met1‐linked chains have been reviewed recently 27, 28.

However, a Met1‐specific DUB had not been described. In 2013, the Cordes group and our lab reported a Met1‐specific hydrolase activity in the previously unannotated DUB of the ovarian tumour (OTU) family, FAM105B 29, 30. Rivkin *et al*. named the protein Gumby, while we named it, and will here refer to it, as OTULIN (OTU domain DUB with LINear linkage specificity, HNGC approved gene symbol). Below, we summarise what has been learned about OTULIN's mechanism, specificity and regulation, and we highlight how the discovery of OTULIN has confirmed many results and opened new avenues to understanding Met1‐polyubiquitin signalling.

## The nature of Met1 linkages and discovery of OTULIN

Ubiquitin linkages are most commonly formed as isopeptide bonds between the C‐terminal Gly76 of a distal ubiquitin moiety and the Nε amino group of a Lys residue. In contrast, LUBAC forms peptide bonds between the ubiquitin C terminus and the α‐amine of Met1 (Fig. 1B). Met1 is spatially close to Lys63, and both chain types behave similarly in solution studies, adopting multiple conformations (Fig. 1B) 16, 31, 32. Despite their similar structure, Met1‐ and Lys63‐linked polyubiquitin chains are distinguished by UBDs 15, 16, 19, 33.

The distinct chemistry of the Met1‐linked peptide bond (Fig. 1B) has important consequences for how these chains are hydrolysed by DUBs, and indeed, many DUBs from all families either do not cleave Met1‐peptide bonds at all, or cleave them with significantly reduced activity 16, 34, 35, 36. An exception to this is the cylindromatosis tumour suppressor (CYLD), a DUB from the ubiquitin‐specific protease (USP) family, which hydrolyses both Lys63 and Met1 linkages with similar activity 16, 37, 38.

In contrast to ‘normal’ DUBs, OTULIN is unable to hydrolyse isopeptide bonds but specifically targets Met1 linkages within polyubiquitin. We found this activity whilst characterising OTU‐family DUBs 36, following bioinformatic predictions of unannotated OTU domain folds in two human proteins, FAM105A and FAM105B. While FAM105A does not contain catalytic triad residues and is inactive, FAM105B cleaved Met1‐linked polyubiquitin chains 29. This was surprising as none of the other human OTU DUBs were able to hydrolyse Met1 linkages 36, thus suggesting a unique mechanism of linkage specificity. In parallel, Cordes and colleagues identified OTULIN in a forward genetic screen for neuronal phenotypes in mice 39, and structural, biochemical and mass‐spectrometry studies of the uncharacterised gene revealed its Met1 linkage specificity and involvement in LUBAC and Wnt signalling processes (see below) 30.

## Understanding Met1 linkage specificity of OTULIN

**Figure 2:**
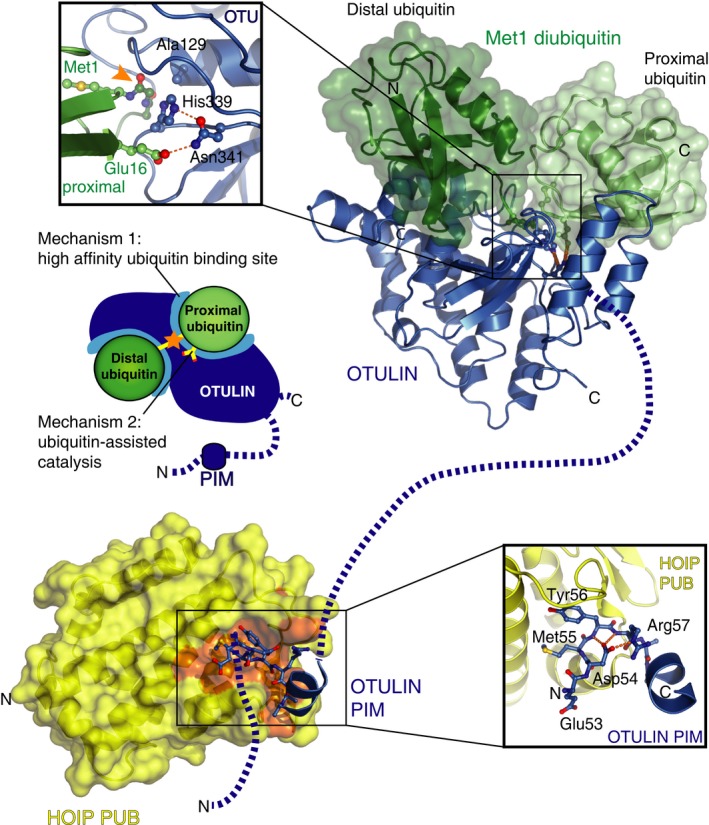
Structural insights into mechanisms of OTULIN. OTULIN is shown in blue with its N terminus (bottom left corner) and disordered regions as dotted lines. The catalytic domain (top right) bound to Met1‐linked diubiquitin (green surface) reveals OTULIN's specificity towards this linkage type. Left inset: close‐up view of the catalytic centre of OTULIN showing the catalytic triad (Cys129 mutated to Ala in the structure, His339, Asn341) coordinated by Glu16 of the proximal ubiquitin moiety. (Middle) Schematic representation of OTULIN specificity for Met1‐linked polyubiquitin. (Bottom left) Structure of the OTULIN PIM (blue) bound to the HOIP PUB domain (yellow surface). (Bottom right) Close‐up view of the HOIP–OTULIN PIM interaction, with the OTULIN PIM shown in ball and stick representation.

Biochemical and structural analysis revealed the molecular basis for OTULIN's specificity (Fig. 2). Like all other Cys protease DUBs, OTULIN belongs to the papain superfamily 40, and contains a catalytic triad with a Cys as reactive nucleophile that attacks the carbonyl of the incoming scissile bond. The catalytic Cys is deprotonated by a His residue, which in turn is oriented and stabilised by an Asp or Asn residue.

The ‘apo’ unbound structure of OTULIN revealed that its catalytic His (His339) was unable to coordinate the catalytic Cys129, as it was held in an inhibited conformation by a non‐catalytic Asp residue (Asp336) 29, 30. Inactive or autoinhibited configurations have been observed in unliganded DUBs previously, and this is usually resolved when ubiquitin binds (for example 41, 42, 43). Interestingly, monoubiquitin binding to OTULIN did not induce rearrangement of the active site into a catalytically competent configuration 30, and monoubiquitin‐based suicide probes, where the C terminus of ubiquitin is replaced with a Cys‐reactive group 44, were unable to react with OTULIN, confirming that the catalytic triad in OTULIN was incompetent 29.

However, these structures did not explain how OTULIN hydrolysed Met1‐polyubiquitin exclusively. This revelation came from structures of inactive OTULIN C129A (where the catalytic Cys was mutated to Ala) bound to Met1‐linked diubiquitin 29, 30 (Fig. 2, top right). The complex structures revealed extensive contacts between OTULIN and both distal and proximal ubiquitin moieties, placing the scissile bond across the active site of OTULIN. Indeed, OTULIN C129A binds Met1‐linked diubiquitin with 150 nm affinity, a 100‐fold greater affinity compared to topologically similar Lys63‐linked diubiquitin. Other linkage types were unable to bind OTULIN C129A 29. The fact that OTULIN has evolved a specific high‐affinity binding site for Met1‐linked diubiquitin partly explains OTULIN's exquisite linkage specificity.

More strikingly, we found that OTULIN was directly activated by the bound diubiquitin. Placement of the proximal ubiquitin moiety in its binding site on OTULIN inserted a ubiquitin Glu side chain, Glu16, directly into the active site of OTULIN (Fig. 2, top left). This ubiquitin Glu has multiple roles. It sterically interferes with the inhibitory Asp336 and pushes the catalytic His339 into an active conformation where it deprotonates the catalytic Cys129. Moreover, ubiquitin Glu16 also directly coordinates the third catalytic residue, Asn341, further organising the catalytic triad (Fig. 2, top right). Consistently, mutation of ubiquitin Glu16 to Ala reduced the catalytic efficiency of OTULIN by 240‐fold without significantly affecting binding of the substrate to the enzyme (similar *K*_d_ and only a threefold reduction in *K*_m_) 29.

The observed ubiquitin‐assisted catalysis, whereby Met1‐linked polyubiquitin facilitates its own cleavage by remodelling of the OTULIN catalytic triad, further explains the exquisite linkage‐specificity of OTULIN (Fig. 2). While the distal ubiquitin in a diubiquitin is identical for each chain type, the proximal ubiquitin presents to the binding partner a unique molecular environment depending on the linkage site, which in the case of OTULIN is utilised to play a part in the catalytic mechanism of the enzyme. Even binding of structurally similar Lys63‐linked diubiquitin leads to a slight rotation of the proximal ubiquitin, such that Glu16 is no longer able to coordinate the catalytic triad.

The OTULIN mechanism of ubiquitin‐assisted catalysis is the first of its kind observed for DUBs, but conceptually similar mechanisms have been described for assembly of Lys11‐linked polyubiquitin by UBE2S 45 and for the NEDD8‐modification of SCF (Skp1, Cullin1, F‐box‐containing) E3 ligase complexes 46. It is becoming increasingly clear that ubiquitin, traditionally viewed as a platform for recognition by binding partners, can also have a more active role in regulating enzyme function.

OTULIN's mechanism requires it to cleave a ubiquitin–ubiquitin linkage, and hence it is unable to cleave the proximal ubiquitin, i.e. a ubiquitin–substrate linkage, since a substrate would be unable to activate OTULIN (Fig. 1A). Hence, proteins targeted by OTULIN will remain (mono)ubiquitinated, and to fully remove (poly)ubiquitin from them, other DUBs need to be involved. Alternatively, the remaining ubiquitin could be extended by the action of E3 ligases with another linkage type (‘ubiquitin chain editing’), resulting in a distinct ubiquitin signal (Fig. 1A).

But why does OTULIN need to be so remarkably specific? Firstly, OTULIN's role as a peptidase requires an additional safety mechanism. Most DUBs are isopeptidases furnished with weak, or no, activity towards peptide bonds – hence they do not present a danger for the cellular protein pool. OTULIN, on the other hand, must ensure that it does not cleave non‐ubiquitin peptide bonds – this is prevented by the observed dual mechanism of specificity described above.

Secondly, substrate‐assisted activation of OTULIN may prevent oxidation of the catalytic Cys, which in other DUBs can undergo reversible and irreversible oxidation 47, 48, 49. Oxidative bursts regulate signalling processes, including the NF‐κB response 50, but may not affect OTULIN function.

Finally, it is now clear that Met1‐polyubiquitin signalling requires tight independent regulation. The known physiological roles of LUBAC and OTULIN clearly show their involvement in crucial cellular decision processes, such as determining the response to infection and the onset of cell death (see below). The requirement for an exquisitely specific E3 ligase and DUB may have arisen from the necessity to tightly control such processes. Indeed, as discussed below, LUBAC and OTULIN form a complex and their activities are likely to be interdependent.

## Met1‐linked ubiquitination in cells

There are at least two cellular sources of Met1‐linked polyubiquitin. First, there are the *UBB* and *UBC* gene products, which encode, in humans, defined chains of three and nine ubiquitin moieties, respectively. This generation of polyubiquitin is conserved in evolution, with varying copy‐numbers per gene in different organisms. The second source of Met1‐linked polyubiquitin is by LUBAC, expression of which is restricted to chordates. Despite these clear sources, mass‐spectrometry or Met1 linkage‐specific antibodies 51 hardly detect any Met1‐linked polyubiquitin in cell lysates. This suggests that cellular DUB activity is efficient at removing Met1‐linked chains from cells. Indeed, free ubiquitin chains such as those generated from *UBB* and *UBC* are highly unstable, in part due to the action of USP5, a highly conserved DUB that recognises and hydrolyses unattached chains 6.

OTULIN is only present in organisms that contain LUBAC, and interacts with LUBAC (see below), suggesting roles of OTULIN in LUBAC‐mediated processes rather than ubiquitin processing functions. OTULIN overexpression removes the residual amounts of Met1 linkages in cells 29. Interestingly, overexpression of inactive OTULIN C129A dramatically increases Met1 linkages in cells, and the increase in Met1 linkages is significantly higher compared to what can be achieved by expressing LUBAC 29. OTULIN's ability to modulate the quantity of Met1 linkages in cells enabled further functional characterisation of this chain type. This has cemented known roles for this chain type in the context of inflammation, infection, and cell death, but also implicated Met1‐linked chains in developmental processes and Wnt signalling.

## OTULIN regulates cytokine responses and inflammation

**Figure 3:**
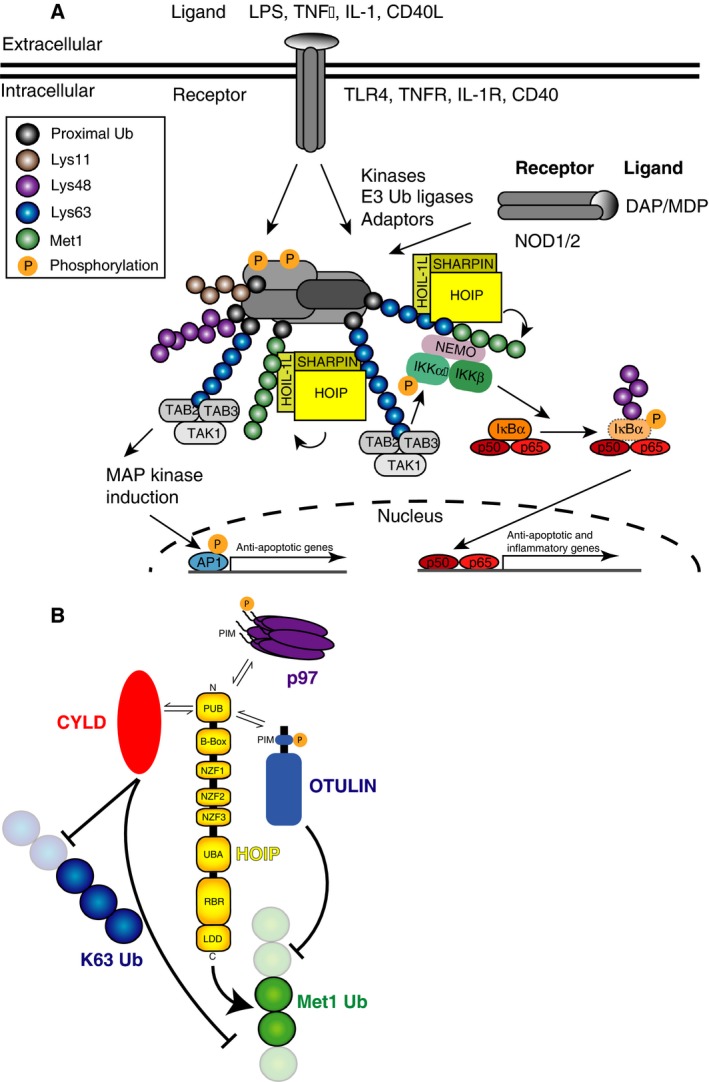
OTULIN in the context of inflammatory signalling. (A) Role of Met1‐linked chains in canonical NF‐κB signalling. Intracellular or extracellular activation results in recruitment of E3 ubiquitin ligases, kinases and various adaptors, cumulating in complexes that contain different types of post‐translational modifications. Polyubiquitin chains serve as recruitment platforms for additional complexes that mediate signalling outcomes. Lys63 chains recruit the TAK1 complex via TAB2 or TAB3, and TAK1 activation leads to mitogen‐activated protein kinase (MAPK) activation and primes IKK for autoactivation. In addition, Lys63‐linked chains recruit LUBAC, which assembles Met1‐linked chains onto Lys63‐linked chains or onto substrates directly, recruiting the IKK complex via NEMO, and contributing to activating the IKK complex. Activated IKK phosphorylates IκBα, which is subsequently degraded. This releases NF‐κB to translocate to the nucleus and act as a transcription factor. (B) The HOIP PUB domain serves as a hub for the recruitment of p97 and the DUBs OTULIN and CYLD. Both CYLD and OTULIN could counteract the ligase activity of LUBAC through cleaving Met1‐linked polyubiquitin chains. In addition to cleaving Met1‐linked chains, CYLD is also able to hydrolyse Lys63‐linked chains. The recruitment of OTULIN is regulated by phosphorylation of invariant Tyr56 in the OTULIN PIM. Kinases/phosphatases that regulate this interaction are unknown, and likewise the mechanism and regulation of CYLD association is also unknown. The role of the HOIP–p97 interaction is less clear as is the timing and recruitment of such a complex.

Met1‐linked ubiquitin chains regulate inflammation as they constitute an important signal during canonical activation of the transcription factor NF‐κB following cytokine stimulation 10, 27, 52 (Fig. 3A).

In resting cells, NF‐κB is sequestered in the cytosol by inhibitor of κB (IκB). Stimulation of cytokine receptors such as tumour necrosis factor (TNF) receptor‐1, or pattern‐recognition receptors such as TLR4, leads to activation of two ubiquitin‐dependent kinase complexes. The TAK1 complex binds to Lys63‐linked chains via its subunits TAB2 or TAB3 and contributes to activation of the IκB kinase (IKK) complex, which itself binds preferentially Met1‐linked chains through its subunit IKKγ/NEMO 53, 54. The IKK complex phosphorylates IκB, leading to IκB ubiquitination with Lys48‐linked polyubiquitin, and degradation by the proteasome. Released NF‐κB translocates into the nucleus to initiate transcription of a large number of proinflammatory genes 55, 56 (Fig. 3A).

Whether and how Lys63‐ and Met1‐linked chains are both required and coordinate the activation of NF‐κB has been a matter of intense debate. In 2013, Cohen and colleagues elegantly demonstrated that LUBAC binds Lys63‐linked chains, and that the chains that can be found on receptor‐proximal proteins (including MyD88 and IRAKs) are in fact polymers containing both Lys63‐ and Met1‐linked chains 57. This discovery of heterotypic or ‘hybrid’ ubiquitin chains reconciles many findings in NF‐κB activation.

OTULIN played a major role in the discovery of mixed linkage chains, as it was used as an *in vitro* tool to reveal the presence of Met1‐linked chains on polyubiquitin ‘smears’. While OTULIN removed predominantly the higher molecular mass chains on immunoprecipitated, polyubiquitinated substrates, a different DUB with high specificity for Lys63‐linked chains, AMSH 58, cleaved the entire polyubiquitin from a substrate. AMSH is unable to cleave Met1‐linked chains, yet Met1 linkages were clearly present in the polyubiquitin smear, revealing that Met1‐linked chains had been attached to pre‐existing Lys63‐polyubiquitin, rather than to the substrate itself. Using linkage‐specific DUBs as tools to understand chain architecture is a method we termed ubiquitin chain restriction (UbiCRest) analysis 59, and this technique holds great promise for gaining further insight into ubiquitin signalling.

How important is the Met1‐polyubiquitin portion of mixed‐linkage chains in NF‐κB activation? Overexpression of OTULIN, and hence removal of all Met1 linkages from cells, blocks NF‐κB translocation to the nucleus upon TNF stimulation and reduces NF‐κB target gene expression. Mechanistically, OTULIN overexpression abrogated association between NEMO and polyubiquitinated RIPK1 29, a key target of ubiquitination in this pathway that is also modified with multiple chain types including Lys63 and Met1 linkages 22. This indicates that key signalling complexes are no longer able to form when OTULIN removes Met1 linkages 29. However, the consequences of OTULIN knockdown were not as dramatic, merely showing delays in NF‐κB activation, and overall mild defects in the signalling cascades 29, 60. Together, this confirmed the prevalent view that in TNF signalling Met1‐linked chains are important for signal strength but not essential to initiate a response 20, 22, 23, 24.

Cytokines such as IL‐1β or stimulation of pattern‐recognition receptors mimicking pathogen infection of cells seem to be more reliant on Met1 linkages, and OTULIN, for signal transduction. Nucleotide binding and oligomerization domain (NOD)‐containing protein pattern recognition receptors sense bacterial pathogens by detecting their peptidoglycan coat, and trigger downstream signalling cascades to orchestrate the inflammatory response 61. Following NOD2 activation, RIPK2 is ubiquitinated by multiple E3 ligases including X‐linked inhibitor of apoptosis (XIAP), which in turn recruits LUBAC to mediate assembly of Met1‐linked ubiquitin chains 62, 63. Destabilisation of LUBAC by loss of SHARPIN impaired NF‐κB activation and the production of the inflammatory cytokines, TNF and IL‐6 62. In contrast, depletion of OTULIN enhanced the activation of NF‐κB in response to NOD2 stimulation 64.

## OTULIN interacts with LUBAC

The tight links between OTULIN and processes known to be regulated by LUBAC, were further consolidated by findings that LUBAC and OTULIN interact directly (Figs 2 and 3B). OTULIN was precipitated with other LUBAC components in SHARPIN immunoprecipitations 29 and mass‐spectrometric and biochemical analysis showed that OTULIN binds to the catalytic subunit of LUBAC, HOIP 30, 60. HOIP is the largest component of LUBAC, and consists of a C‐terminal E3 ligase module (RBR domain and LDD), a middle section with ubiquitin and substrate binding domains 21, 57, 65, 66, as well as domains for interacting with the other LUBAC components 65, and an N‐terminal region containing a PUB (peptide:N‐glycanase/UBA‐ or UBX‐containing proteins) domain and B‐box domain of unknown function (Figs 2, bottom and 3B).

Mapping analysis showed that the N‐terminus of OTULIN bound to the PUB domain in HOIP. PUB domains had been identified in UBXN6 and PNGase, and bind the hexameric AAA+ ATPase p97 (also known as VCP or cdc48 in yeast) 67. Characterisation of the binary interaction revealed that the PUB domain bound the last three residues of p97, and this short motif, (Asp)‐Leu‐Tyr‐Gly‐(COO^−^), was termed PUB‐interaction motif (PIM) or p97 binding site (PBS) 68, 69, 70. PIM peptides form a tight hook‐like structure and bind PUB domains with low micromolar affinity (3–10 μm) 68, 69.

A similar PIM in OTULIN was identified in the highly conserved, flexible N terminus (aa 55–57, Asp54‐Met55‐Tyr56‐Arg57‐Ala58) 60, 71, and this internal PIM binds the HOIP PUB domain with high affinity (*K*_d_ ~ 200 nm), 40 times better than a p97‐derived PIM peptide. Moreover, the OTULIN PIM is specific for HOIP and does not interact with PUB domains of PNGase or UBXN6 71. Crystal structures of the HOIP PUB domain in complex with PIM peptides from OTULIN and p97 revealed the molecular basis for the interaction and PUB domain specificity 60, 71 (Fig. 2, bottom left). Subtle differences in the PIM pocket of the PUB domain in HOIP explain how it can interact preferentially with the internal PIM in OTULIN as opposed to the C‐terminal PIM in p97. Indeed, HOIP interacts specifically with residues extending from the internal PIM, namely Ala58 (Fig. 2, bottom right), and does not recognize the free C‐terminus of the p97 PIM. Mutation of HOIP to enable recognition of the C‐terminus improves its binding to p97 69, 71.

Together, the biophysical data in Schaeffer *et al*. and Elliott *et al*. reveal the molecular basis for the high affinity interaction of the LUBAC complex with OTULIN, indicating how OTULIN can regulate LUBAC targets 29, 60, 71. Interestingly, Takiuchi *et al*. further showed that CYLD also interacts with LUBAC via the HOIP PUB domain (see below) (Fig. 3B) 72. Hence, the PUB domain of HOIP has at least three binding partners, OTULIN, CYLD and p97 60, 71, 72. The p97 PIM is unable to outcompete OTULIN 71, but it is possible that separate pools of p97‐, OTULIN‐ and CYLD‐bound LUBAC complexes coexist. Although the role of p97 in LUBAC‐mediated NF‐κB activation is currently unclear, a recent study has shown that p97 is involved in the degradation of polyubiquitinated IκBα 73.

## Regulating the HOIP‐OTULIN interaction

An interaction between HOIP and OTULIN or CYLD seems strange, since their highly specific yet counteracting activities would generate a futile energy‐consuming circle (Fig. 3B). However, E3 ligases are common DUB interactors 74, and studies of selected pairs have generated interesting concepts of cross‐regulation. For example, the DUB may ensure E3 ligase stability, since E3 ligases tend to autoubiquitinate 75, 76, 77, 78. Conversely, the associated DUB may have reduced stability whilst associated with an E3 ligase, as in the case of USP20–HERC2 79. DUB–E3 interactions may also regulate the ubiquitin chain type attached to substrates, or even regulate the activity of the E3 ligase, as in the case of ataxin3–CHIP 80.

Importantly, knockdown of OTULIN leads to Met1‐autoubiquitination of HOIP, HOIL1L and SHARPIN, in particular after stimulation 64, suggesting that one role of OTULIN is to keep LUBAC in an unubiquitinated state. While autoubiquitination does not appear to be autoinhibitory 29, 64, it may regulate LUBAC's association with signalling complexes. Indeed, it is less clear whether the OTULIN–LUBAC complex travels together, e.g. to the TNF receptor. SHARPIN immunoprecipitated both OTULIN and the TNF receptor 29, and one report found overexpressed OTULIN being part of the TNF receptor signalling complex 60. However, we have been unable to detect endogenous OTULIN in TNF receptor signalling complex precipitates. It is hence possible that OTULIN is not co‐recruited to all sites of LUBAC action, such as the activated TNF receptor, alleviating the issue of antagonising activities.

Why would OTULIN dissociate from HOIP? A key residue in the OTULIN PIM is the invariant Tyr56, and phospho‐proteomic analysis has repeatedly found this residue to be phosphorylated, for example upon activation of MET‐ and EGF‐receptor signalling (see http://www.phosphosite.org/proteinAction.do?id=2470471&showAllSites=true). Consistent with the structure, phosphorylation of Tyr56 prevents interaction between OTULIN and HOIP 60, 71 (Fig. 2, bottom). Furthermore, while OTULIN and the HOIP PUB domain form a gel filtration‐stable complex *in vitro*, the majority of OTULIN in cell lysates does not coelute with LUBAC indicating that OTULIN may be phosphorylated in cells, and dephosphorylation of OTULIN in cell extracts led to coelution of OTULIN with LUBAC 71. Phosphorylation‐dependent regulation of the interaction between OTULIN and HOIP offers an elegant additional layer of control for Met1‐signalling (Fig. 3B). However, the kinases or phosphatases regulating the interaction between OTULIN and HOIP are still unknown.

## OTULIN and linear chains in the context of other NF‐κB regulating DUBs

Ubiquitin‐dependent NF‐κB activation is regulated by a multitude of DUBs, including the OTU family enzyme A20, as well as various USP family enzymes including CYLD.

The OTU family DUB A20 is a master regulator of the NF‐κB response 81, and a plethora of knock‐out and knock‐in models have highlighted its involvement in inflammation 82. However, despite significant efforts, A20′s mechanism of action is not fully understood. The initial model that A20 itself selectively removes Lys63 chains from target proteins via its OTU DUB domain, and then adds Lys48‐linked chains via C‐terminal A20‐like ZnF domains 83, has been substantially extended by findings that A20 interacts with various E3 ligases 84, 85, and that the seventh and last A20‐ZnF domain, which is crucial for A20 function, constitutes a Met1 linkage‐specific UBD 17, 18. The OTU DUB domain itself has poor activity to cleave polyubiquitin, is unable to hydrolyse Met1 linkages and prefers Lys48 rather than Lys63 linkages *in vitro* 36. It is debated whether its activity is required 86, 87. Hence, how exactly A20 edits polyubiquitin in cytokine signalling requires further study.

CYLD was originally identified as the tumour suppressor mutated in familial cylindromatosis 88 and found to affect NF‐κB signalling 89, 90, 91. Importantly, CYLD is the only known USP DUB to hydrolyse Met1‐ and Lys63‐linked polyubiquitin with some preference 16, 37, 38, providing it with suitable activity to regulate NF‐κB. CYLD‐deficient mice have less pronounced phenotypes as compared to A20 30, 92, and the gene is not substantially induced by NF‐κB. Interestingly, CYLD also binds the HOIP PUB and B‐box domains despite lacking a discernible PIM. It will be interesting to see how CYLD and LUBAC interact, and how Met1‐sigalling is differentially (competitively?) regulated by CYLD and OTULIN. Indeed it is intriguing that OTULIN, A20 and CYLD either bind or hydrolyse Met1 linkages with some degree of specificity.

Several other USP domain DUBs were suggested to act on one or several ubiquitinated proteins in the NF‐κB activating cascade, including USP4, which may act on TRAF2 and TRAF6 93, 94, and USP7, which enhances NF‐κB responses by deubiquitinating and stabilising NF‐κB directly 95. USP21 was reported to target RIPK1 and inhibit NF‐κB activity 96, however mice lacking USP21 show no defect in NF‐κB signalling 93. Genetic evidence for regulatory roles is still lacking for the other enzymes, and while there is significant room for regulation by DUBs, the fact that signals such as TNF that lead to strong enrichment of polyubiquitin, may be regulated non‐specifically by overexpressed DUBs. For example, upregulation of a highly active and non‐specific USP enzyme in cancer may dampen the inflammatory response non‐specifically, while the endogenous DUB may not be involved in regulating NF‐κB.

It is clear that the interplay of DUBs regulating NF‐κB is complex and requires further study and more detailed genetic models. At the same time, NF‐κB is only one of many signalling pathways regulated by non‐degradative chains, and their involvement in other pathways is easy to imagine.

## New pathways regulated by OTULIN and involving Met1‐ubiquitination

Studies of OTULIN led to new insights into Met1 ubiquitin signalling and revealed a new pathway likely regulated by LUBAC/OTULIN and Met1‐linked chains. Rivkin *et al*. originally identified two distinct mutations in the *Otulin* gene, W96R and D336E, in a forward genetic screen for neuronal phenotypes in mice 30, 39. The mutations are located in the catalytic domain and inactivate OTULIN 30, likely by disrupting both activity and binding to Met1‐linked chains 29. *Otulin* mutant mice are embryonically lethal due to angiogenesis defects 30, and both point mutations in *Otulin* affected canonical Wnt signalling in mice and human cells. Moreover, a reported interaction of OTULIN and dishevelled‐2 (DVL2) could be confirmed, suggesting a role of Met1‐linked ubiquitination in Wnt signalling 30, 72.

CYLD also regulates Wnt signalling at the level of DVL2 97. However, while CYLD knockdown enhances β‐catenin translocation to the nucleus, OTULIN mutant mice expressing a Wnt reporter showed reduced pathway activation 30. It will be interesting to learn about targets, receptors and consequences for Met1‐ubiquitination in Wnt signalling and animal development. This is also important in the light of recently published HOIP knockout mice 98 that are embryonically lethal at an earlier stage (E10.5 as compared to E12.5–14 for *Otulin* mutant mice) stage, and the observed lethality was associated with defects in cell death pathways.

## Perspectives

Despite its recent discovery significant, we have already gained insights into OTULIN mechanism, regulation, and cellular roles. Now, model systems are required to examine OTULIN's physiological roles in cells and organisms. Since OTULIN does not have functionally similar orthologues, knock‐out studies should enable a better understanding of Met1‐signalling in inflammation, immunity and development. Due to the embryonic lethality of OTULIN in mice, tissue specific or inducible mouse models could be beneficial.

It would also be interesting to develop knock‐in models. Nevertheless, simply mutating the catalytic Cys in DUBs to Ala or Ser, as used for A20 86, 87, is problematic, as this potentially converts the DUB domain to a high‐affinity UBD; OTULIN C129A binds Met1‐diubiquitin with nanomolar affinity, and interferes with NF‐κB signalling in artificial ways 29. Preferable would be a mutant that abrogates both activity and also polyubiquitin binding, as reported for the OTULIN W96R or W96A mutant 29, 30. A further interesting mutation could be OTULIN Y56F, which is unable to interact with LUBAC and could report on the importance of OTULIN‐LUBAC interaction *in vivo*.

OTULIN function is likely regulated by post‐translational modifications, including phosphorylation of Tyr56, but the enzymes mediating this phosphorylation event are unknown, and it is not clear how dynamic this process is. A related area of great potential is the identification of further binding partners for OTULIN. Rivkin *et al*. commented on PDZ‐containing proteins that interacted with OTULIN in pull‐down assays, and removal of the OTULIN C terminus abrogated these interactions 30. Gel filtration profiles indeed suggest that OTULIN participates in alternative complexes 71. Such signalling complexes could give more insights into the role of OTULIN and Met1‐linked chains in cells.

FAM105A is highly similar to OTULIN (41% identity) and contains an OTU domain that may well bind ubiquitin, but FAM105A lacks catalytic residues. FAM105A and OTULIN are located adjacent to each other on chromosome 5p15.2 and likely arose from an early gene duplication event; *Ciona* species have only one (active) OTULIN gene. Overexpression screens identified Fam105A as a novel proapoptotic gene 99, but the function of this protein has not been studied.

Finally, it will be interesting to study disease association of OTULIN. The functionally similar DUBs A20 and CYLD are both tumour suppressors, and while some functionally disabling mutations are reported in the COSMIC database (http://cancer.sanger.ac.uk/cosmic/gene/analysis?ln=FAM105B) particular cancer types that depend on loss of OTULIN have not been identified. Moreover, OTULIN is located in a region of the genome lost in Cri‐du‐Chat disease (http://ghr.nlm.nih.gov/condition/cri-du-chat-syndrome), a developmental disorder characterized by intellectual disability and delayed development, small head size (microcephaly), and distinctive facial features. The latter correlate with developmental phenotypes in *Otulin* knockout mice 39, with the difference that heterozygous mice are normal, while loss of just one 5p region causes the human disease. Nonetheless, the clear roles of OTULIN in development open a new perspective upon Met1 signalling beyond inflammation and infection.

It is clear that OTULIN was a missing piece in the puzzle of Met1‐polyubiquitin signalling, yet much more remains to be learned about its regulation and cellular roles.

## Competing financial interests

DK is part of the DUB Alliance that includes Cancer Research Technology and FORMA Therapeutics, and is a consultant for FORMA Therapeutics.

## Author contributions

PRE and DK wrote the Review.
